# Increased Muscular 5α-Dihydrotestosterone in Response to Resistance Training Relates to Skeletal Muscle Mass and Glucose Metabolism in Type 2 Diabetic Rats

**DOI:** 10.1371/journal.pone.0165689

**Published:** 2016-11-10

**Authors:** Naoki Horii, Koji Sato, Noboru Mesaki, Motoyuki Iemitsu

**Affiliations:** 1 Faculty of Sport Health Science, Ritsumeikan University, Kusatsu, Shiga, Japan; 2 Graduate School of Human Development and Environment, Kobe University, Kobe, Hyogo, Japan; 3 University of Tsukuba, Tsukuba, Ibaraki, Japan; Universidade do Estado do Rio de Janeiro, BRAZIL

## Abstract

Regular resistance exercise induces skeletal muscle hypertrophy and improvement of glycemic control in type 2 diabetes patients. Administration of dehydroepiandrosterone (DHEA), a sex steroid hormone precursor, increases 5α-dihydrotestosterone (DHT) synthesis and is associated with improvements in fasting blood glucose level and skeletal muscle hypertrophy. Therefore, the aim of this study was to investigate whether increase in muscle DHT levels, induced by chronic resistance exercise, can contribute to skeletal muscle hypertrophy and concomitant improvement of muscular glucose metabolism in type 2 diabetic rats. Male 20-week-old type 2 diabetic rats (OLETF) were randomly divided into 3 groups: sedentary control, resistance training (3 times a week on alternate days for 8 weeks), or resistance training with continuous infusion of a 5α-reductase inhibitor (n = 8 each group). Age-matched, healthy nondiabetic Long-Evans Tokushima Otsuka (LETO) rats (n = 8) were used as controls. The results indicated that OLETF rats showed significant decrease in muscular DHEA, free testosterone, DHT levels, and protein expression of steroidogenic enzymes, with loss of skeletal muscle mass and hyperglycemia, compared to that of LETO rats. However, 8-week resistance training in OLETF rats significantly increased the levels of muscle sex steroid hormones and protein expression of steroidogenic enzymes with a concomitant increase in skeletal muscle mass, improved fasting glucose level, and insulin sensitivity index. Moreover, resistance training accelerated glucose transporter-4 (GLUT-4) translocation and protein kinase B and C-ζ/λ phosphorylation. Administering the 5α-reductase inhibitor in resistance-trained OLETF rats resulted in suppression of the exercise-induced effects on skeletal muscle mass, fasting glucose level, insulin sensitivity index, and GLUT-4 signaling, with a decline in muscular DHT levels. These findings suggest that resistance training-induced elevation of muscular DHT levels may contribute to improvement of hyperglycemia and skeletal muscle hypertrophy in type 2 diabetic rats.

## Introduction

In 2014, 9% of adults 18 years and older around the world had diabetes, and diabetes was the direct cause of 1.5 million deaths [1, 2]. The beneficial effects of regular aerobic exercise for type 2 diabetics have been well established, which include restoration of glycemic control and reduced insulin resistance without muscle hypertrophy [3–6]. Additionally, regular resistance exercise reduces fasting glucose and glycosylated hemoglobin (HbA1c) of type 2 diabetic patients in randomized, controlled studies [3–6]. On a molecular level, an increase in expression and translocation of glucose transporter 4 (GLUT-4) in the skeletal muscle following resistance training contributes to improvement of glycemic control [7]. Furthermore, decreased skeletal muscle mass is associated with a deterioration of insulin resistance [8], thus, the increase in muscle mass in response to resistance training may participate in improvement of glycemic control.

Sex steroid hormones, especially testosterone and 5α-dihydrotestosterone (DHT), play an important role in regulating muscular energy metabolism and skeletal muscle hypertrophy [9, 10]. We previously showed that dehydroepiandrosterone (DHEA), a steroid hormone precursor, is metabolized to testosterone and DHT in cultured skeletal muscle, suggesting that skeletal muscle is capable of locally synthesizing sex steroid hormones [11, 12]. Moreover, in our previous study on obese diabetic rats, chronic DHEA supplementation increased muscular DHT levels and upregulation of muscular glucose metabolism via elevated GLUT-4 translocation and skeletal muscle hypertrophy [13]. In type 2 diabetic patients and hyperglycemic obese and type 2 diabetic rats, blood and/or muscular levels of DHEA and its sulfate derivate (DHEA-S) are reduced compared to healthy controls [13, 14]. Our recent study showed that chronic resistance exercise elevated muscular DHT levels, which correlated with training-induced skeletal muscle hypertrophy in healthy older patients [15]. Accordingly, these steroid hormones may be a part of the mechanism through which resistance exercise leads to better glycemic control with skeletal muscle hypertrophy in patients with type 2 diabetes. However, it remains unclear whether chronic resistance exercise-induced increases in muscle sex steroid metabolism can contribute to improvement of muscular glucose metabolism and skeletal muscle hypertrophy in type 2 diabetics.

Therefore, the aim of this study was to investigate whether 8 weeks of resistance training can enhance muscle steroidogenesis and whether increased sex steroid hormone levels in skeletal muscle contributes to improvement of muscular glucose metabolism and skeletal muscle hypertrophy in type 2 diabetic rats. To test our hypothesis, we allowed Otsuka Long-Evans Tokushima Fatty (OLETF) diabetic rats to participate in resistance exercise with and without chronic infusion of a 5α-reductase inhibitor, which results in decreased conversion of testosterone to DHT, and evaluated the effects of resistance exercise-induced increases in sex steroid hormones on glucose metabolism via GLUT-4 regulated signaling and skeletal muscle mass.

## Methods

The ethical approval for this study was obtained from the Committee on Animal Care at the Ritsumeikan University. Male OLETF and Long-Evans Tokushima Otsuka (LETO) rats (6 weeks old) were obtained (Japan SLC, Shizuoka, Japan) and cared for according to the *Guiding Principles for the Care and Use of Animals*, based on the Declaration of Helsinki. The rats were housed individually in an animal facility under controlled conditions (12:12-h light:dark cycle, with the light period being from 8:00 A.M. to 8:00 P.M.), and were given access to water and fed normal chow (CE2; CLEA Japan, Tokyo, Japan). After 14 weeks, the 20-week-old OLETF rats were randomly divided into three groups (n = 8 each group): sedentary control (Con), resistance training (RT), or resistance training with continuous infusion of 5α-reductase inhibitor (RT+In). The 5α-reductase inhibitor (dutasteride; Sigma, Steinheim, Germany) was administrated continuously at 0.15 μL per h for 8 weeks via an implanted osmotic mini pump in subcutaneous adipose tissue (Model 2006; Alzet, Cupertino, CA) [16, 17]. The inhibitor (2 mg/kg) was dissolved into sesame oil and 200 μL was loaded into the pump [16]. Additionally, nondiabetic, healthy, and age-matched LETO rats (n = 8) were used as controls. Post-treatment experiments in trained rats were performed 48 h after the last exercise session to avoid acute effects of exercise. All rats were fasted for 12 h and after measuring body weight, blood samples were obtained from the abdominal aorta under general anesthesia. After sacrifice, the heart, epididymal fat, soleus, gastrocnemius, and plantaris muscles were resected quickly, rinsed in ice-cold saline, weighed, and frozen in liquid nitrogen. The gastrocnemius muscle was used to evaluate the expressions of steroidogenic enzymes and hormones levels and perform histochemical analysis of the skeletal muscle, thus providing a steroidogenic status of the tissue.

### Resistance training protocol

Resistance training was performed 3 days a week on alternate days for 8 weeks using a ladder with length 1.1 m, grid step 2.0 cm, and incline of 80° according to a previous study [18]. The rats in training groups climbed the ladder for 3 sets of 4 repetitions each, and were allowed to rest between sets for 1 min.

### Immunoblot analysis

Western blot analysis was performed as previously described [16, 19]. Briefly, muscle proteins (40 μg) were separated on 10% SDS-polyacrylamide gels, and then transferred to polyvinylidene difluoride (PVDF) membranes (Millipore, Billerica, MA, USA). The membranes were blocked for 1 h with blocking buffer (5% skim milk in phosphate-buffered saline with 0.1% Tween 20) and then incubated for 12 h at 4°C with primary antibodies against– 3β-HSD (sc-30820, Santa Cruz Biotechnology, Dallas, TX, USA), 17β-HSD (sc-26963, Santa Cruz Biotechnology), androgen receptor (sc-816, Santa Cruz Biotechnology), 5α-reductase (#H00006715-D01P, Abnova Corporation, Taipei, Taiwan), serine (Ser)^473^-phosphorylated Akt (#9272, Cell Signaling, Beverly, MA), total Akt (#9272, Cell Signaling), phosphorylated PKC-ζ/λ (#9378, Cell Signaling), or GLUT-4 (#07–1404, Millipore), all diluted to 1:1000 in blocking buffer. β-actin protein (sc-47778, Santa Cruz Biotechnology) was used as an internal control. The membranes were washed three times with PBS-T, and then incubated with a horseradish peroxidase (HRP)-conjugated secondary antibody, which was an anti-rabbit (1:3000 dilution with blocking buffer, GE Healthcare Biosciences, Piscataway, NJ, USA; Cell signaling), an anti-goat (1:3000 dilution with blocking buffer, Santa Cruz Biotechnology), or an anti-mouse (1:3000 dilution with blocking buffer, GE Healthcare Biosciences) immunoglobulins for 1 h at room temperature. After these reactions, the membranes were washed three times with PBS-T. Finally, proteins were detected using an enhanced chemiluminescence plus system (GE Healthcare Biosciences) and visualized on an LAS4000 imager (GE Healthcare Biosciences) [16].

### Preparation of cytosolic and plasma membrane protein fractions

For determination of GLUT-4 translocation, two different cellular fractions were used as previously described [16, 20, 21]. Briefly, the cells were scraped in buffer A containing (in mM) 20 Tris (pH 7.4), 1 EDTA, 0.25 EGTA, 1 DTT, 50 NaF, 25 sodium pyrophosphate, 40 β-glycerophosphate, and 250 sucrose. The resulting homogenates were centrifuged at 400 ×*g* for 15 min to remove debris. The supernatant was centrifuged again at 50,000 rpm for 1 h. The resulting supernatant used as the cytosol protein fraction. Additionally, the pellet from different fractions were solubilized for 1 h at room temperature in buffer B containing 20 mM Tris (pH 7.4), 1 mM EDTA, 0.25 mM EGTA, 2% Triton X-100, 50 mM NaF, 25 μM sodium pyrophosphate, and 40 mM β-glycerophosphate. The homogenate was centrifuged briefly, and the supernatant was spun for 1 h at 50,000 rpm before it was used as the plasma membrane fraction. GLUT-4 protein levels were measured in both the cytosol and membrane fractions. Translocation was evaluated based on the relative abundance of protein levels in these fractions [13, 16].

### Sandwich enzyme immunoassay

The levels of plasma DHEA and plasma DHT, and the levels of DHEA, free testosterone, and DHT in skeletal muscle extracts were determined using a sandwich enzyme-linked immunosorbent assay (ELISA) kit (Assay Designs, Ann Arbor, MI, USA, IBL Hamburg, Germany). The immobilized polyclonal antibodies were raised against DHEA, free testosterone, and DHT, whereas the secondary HRP-coupled antibodies were monoclonal. Optical density at 450 nm was qualified on a microplate reader using xMark microplate spectrophotometer (Bio-Rad Laboratories, Hercules, CA, USA). All samples were assayed in duplicate.

### Fasting glucose and insulin concentrations

Fasting glucose was assessed from the tail vein after the treatment period under overnight fasting conditions. Glucose concentrations were assessed three times from the tail vein using a blood glucose meter (Ascensia, Bayer HealthCare, Tokyo, Japan). Serum insulin concentrations were measured using an ELISA kit (Shibayagi, Gunma, Japan). Optical density at 450 nm was quantitated using a microplate reader. All samples were assayed in duplicate.

### Insulin sensitivity

As an index of insulin sensitivity, quantitative insulin sensitivity check index (QUICKI) was calculated according to previous studies from fasting glucose and insulin concentration [22–24]. QUICKI = 1/[log(I_0_) + log(G_0_)], where I_0_ is fasting insulin (μU/mL), and G_0_ is fasting glucose (mg/dL).

### Histochemical analysis

Gastrocnemius muscle samples frozen for histochemical analysis were sectioned (10 μm thick) in a cryostat at -20°C. The sections were subjected to hematoxylin and eosin staining and measurement of muscle cross-sectional areas (CSA) [25]. At least 5 sections were taken from each sample and at least 10 microscopic fields were examined at ×400 magnification by using Leica DFC425 microscope (Leica Microsystems, Tokyo, Japan). The myocyte scan area was calculated using ImageJ 1.48 software (National Institutes of Health) as previously described [7].

### Statistical analysis

All values are expressed as means ± SEM. Statistical evaluations were performed using one-way ANOVA. A Fisher’s post-hoc test was used to correct for multiple comparisons when analyses revealed significant differences. For ANOVA, P < 0.05 was considered significant. Relationships between muscular DHT level, gastrocnemius muscle mass or GLUT-4 translocation and other factors were determined using Pearson correlation coefficients.

## Results

**Fig 1:**
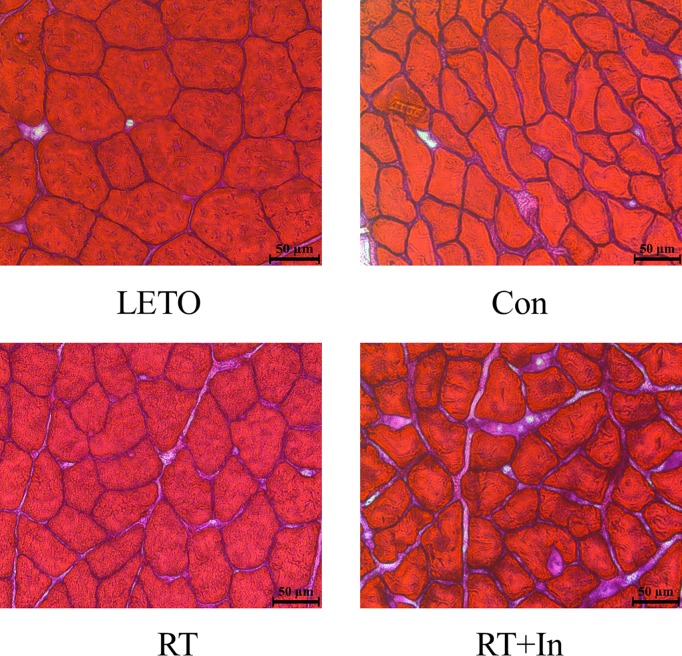
Representative histological images of cross-sectional gastrocnemius muscle, stained with hematoxylin and eosin. Scale bars: 50 μm.

Body weight and epididymal fat mass in the OLETF control rats were significantly higher, and soleus, plantaris, and gastrocnemius muscle mass and CSA in gastrocnemius muscle were significantly lower than those in LETO rats (Tables 1 and 2 and Fig 1). Compared to the OLETF control rats, resistance-trained OLETF rats showed decreased body weight and epididymal fat mass, and increased LV mass, soleus, plantaris, and gastrocnemius muscle mass and CSA in gastrocnemius muscle (Tables 1 and 2 and Fig 1). Compared to LETO rats, OLETF control rats showed significantly higher fasting blood glucose and insulin levels and significantly lower QUICKI values (Table 1). The resistance-trained OLETF rats had significantly decreased fasting glucose levels and significantly increased QUICKI values compared to OLETF control rats (Table 1). However, the OLETF rats that were resistance-trained and received the 5α-reductase inhibitor showed no improvement in fasting blood glucose levels or QUICKI, and no increase in skeletal muscle mass compared to the resistance-trained OLETF rats, but the effects of gastrocnemius CSA and fasting blood glucose levels on resistance training was not completely suppressed (Tables 1 and 2 and Fig 1). Moreover, the resistance training-induced cardiac hypertrophy was not affected by the 5α-reductase inhibitor (Table 1). Average dietary intake over the 8-week experimental period was significantly higher in three OLETF groups than that in the LETO group (Table 1), whereas no significant difference was observed in the dietary intake among the three OLETF groups (Table 1).

**Table 1:** Animal Characteristics.

		OLETF		
	LETO (n = 8)	Con (n = 8)	RT (n = 8)	RT+In (n = 8)
Body weight (g)	480.5±10.5*^†^^‡^	624.9±6.0	588.4±10.6*^‡^	638.4±6.9
Epididymal fat mass (g)	8.77±0.24*^†^^‡^	16.07±1.06	13.04±0.88*^‡^	15.65±1.07
Left ventricle/ body weight (mg/g BW)	1.88±0.07^†^^‡^	1.83±0.09	2.82±0.12*	2.66±0.07*
Fasting glucose (mg/dl)	109.3±5.5*^†^^‡^	350.0±12.5	183.5±19.4*^‡^	290.4±28.0*
Fasting insulin (pmol/l)	1.30±0.11*^†^^‡^	10.88±1.03	10.04±1.07	10.24±1.03
QUICKI	0.438±0.006*^†^^‡^	0.270±0.003	0.297±0.006*^‡^	0.279±0.004
Dietary intake (g/day)	20.6±0.4*^†^^‡^	27.6±0.8	26.7±1.0	26.7±1.1

Values are means ± SE. LETO, healthy sedentary-control group; Con, OLETF-sedentary control group; RT, OLETF-resistance training group, RT+In; OLETF-resistance training + 5α reductase inhibitor group

*P < 0.05 vs. OLETF-sedentary control group

^†^P < 0.05 vs. OLETF-resistance training group

^‡^P < 0.05 vs. OLETF-resistance training + 5α-reductase inhibitor group.

**Table 2:** Muscle mass and cross sectional area.

		OLETF		
	LETO (n = 8)	Con (n = 8)	RT (n = 8)	RT+In (n = 8)
Gastrocnemius/ body weight (mg/g BW)	4.92±0.15*^†^^‡^	3.33±0.08	4.25±0.08*^‡^	3.61±0.12
Soleus/ body weight (mg/g BW)	0.39±0.01*^‡^	0.32±0.01	0.37±0.01*^‡^	0.30±0.01
Plantaris/ body weight (mg/g BW)	0.85±0.02*^‡^	0.66±0.02	0.78±0.03*^‡^	0.71±0.02
Gastrocnemius CSA (μm^2^)	2084±68.3*^†^^‡^	1405±53.2	1787±26.2*^‡^	1572±68.6*

Values are means ± SE. LETO, healthy sedentary-control group; Con, OLETF sedentary control group; RT, OLETF-resistance training group, RT+In; OLETF-resistance training + 5α reductase inhibitor group; CSA; cross-sectional areas

*P < 0.05 vs. OLETF-sedentary control group

^†^P < 0.05 vs. OLETF-resistance training group

^‡^P < 0.05 vs. OLETF-resistance training + 5α-reductase inhibitor group.

### Plasma and tissue levels of sex steroid hormones and expression of steroidogenic enzymes

**Fig 2:**
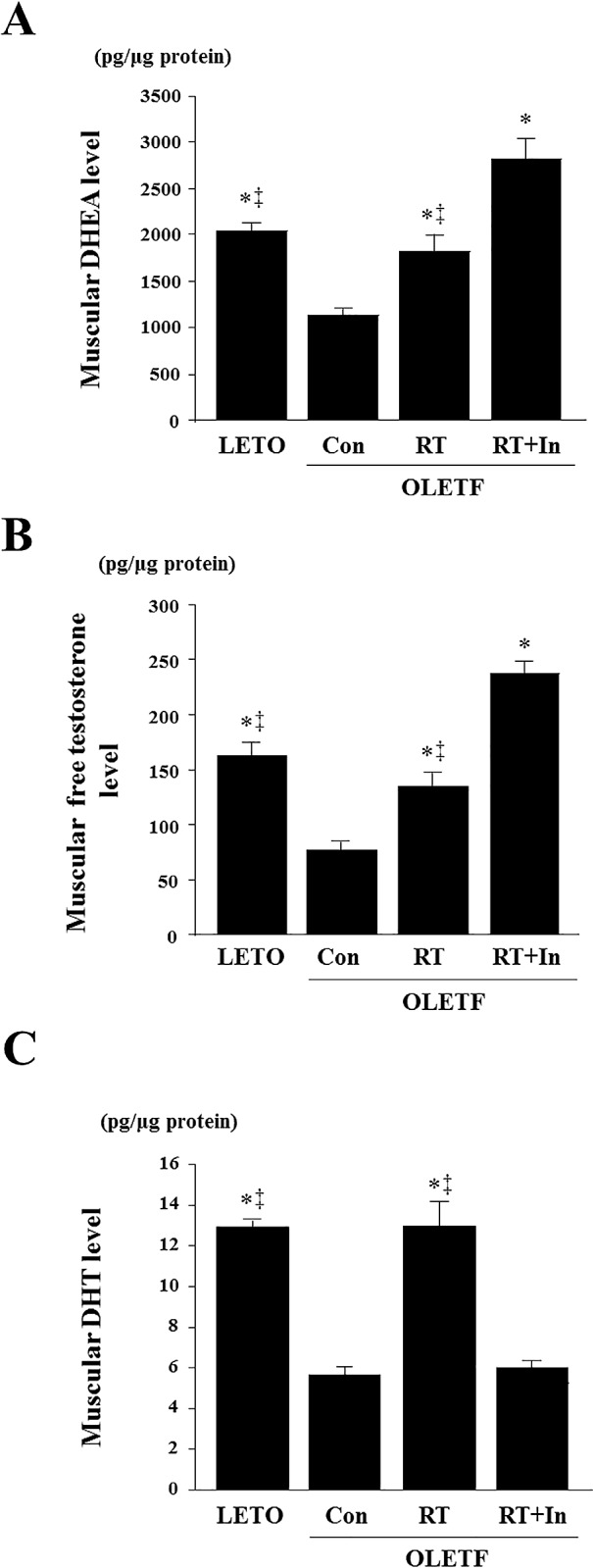
Effects of resistance training on muscular DHEA (A), testosterone (B), and DHT (C) levels in gastrocnemius muscle. Data are expressed as means ± SE. *P < 0.05 vs. OLETF-sedentary control group; ^‡^P < 0.05 vs. OLETF-resistance training group + 5α-reductase inhibitor group.

**Fig 3:**
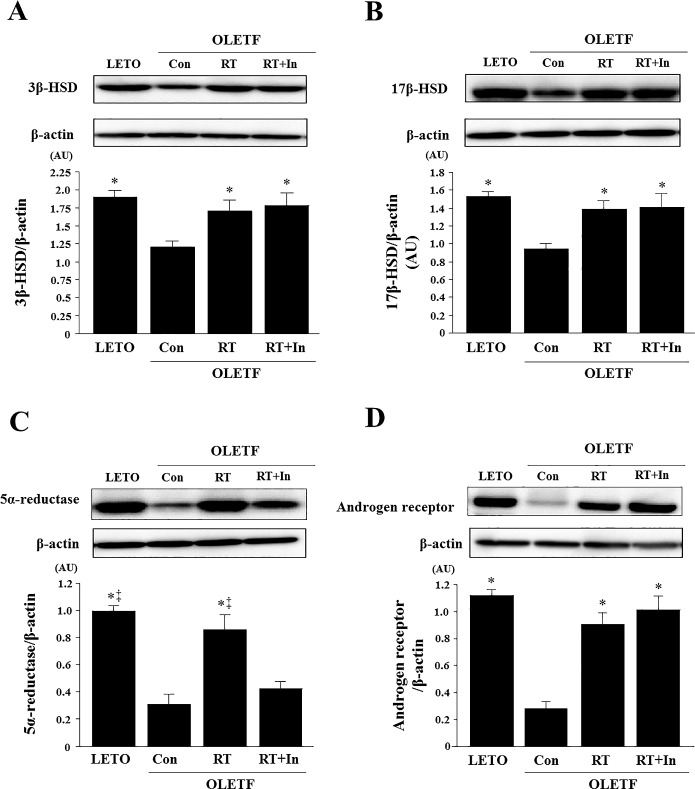
Effects of resistance training on 3β-HSD (A), 17β-HSD (B), 5α-reductase (C), and androgen receptor (D) protein levels in gastrocnemius muscle. Representative immunoblotting images and histograms of 3β-HSD, 17β-HSD, 5α-reductase, and androgen receptor proteins are shown. β-actin protein was employed as an internal control for normalization. AU, arbitrary units. Data are expressed as means ± SE. *P < 0.05 vs. OLETF-sedentary control group; ^‡^P < 0.05 vs. OLETF-resistance training group + 5α-reductase inhibitor group.

Plasma DHEA and plasma DHT levels, and muscle DHEA, free testosterone, and DHT levels were significantly lower in OLETF control rats than in LETO rats, and these levels in plasma and skeletal muscle were significantly elevated in the OLETF rats after resistance training (Table 3, Fig 2). However, the resistance training-induced increase in DHT was suppressed by chronic administration of the 5α-reductase inhibitor (Table 3, Fig 2). Moreover, expression of androgen receptor and steroidogenic enzymes such as 3β-HSD, 17β-HSD, and 5α-reductase were significantly lower in OLETF control rats than in LETO rats (Fig 3); however, resistance training increased expression of these proteins (Fig 3). After administration of the 5α-reductase inhibitor in the resistance-trained OLETF rats, the training-induced increase in 5α-reductase expression was suppressed in the skeletal muscle, but expression of 3β-HSD, 17β-HSD, and androgen receptor was not affected (Fig 3).

**Table 3:** Plasma sex steroid hormone.

		OLETF		
	LETO (n = 8)	Con (n = 8)	RT (n = 8)	RT+In (n = 8)
Plasma DHEA (pg/ml)	4184.7±282.4*^†^^‡^	2429.9±113.9	3482.8±241.5*	3277.6±221.8*
Plasma DHT (pg/ml)	2.03±0.14*^†^^‡^	0.52±0.07	0.99±0.10*^‡^	0.15±0.02*

Values are means ± SE. LETO, healthy sedentary-control group; Con, OLETF-sedentary control group; RT, OLETF-resistance training group, RT+In; OLETF-resistance training + 5α reductase inhibitor group

*P < 0.05 vs. OLETF-sedentary control group

^†^P < 0.05 vs. OLETF-resistance training group

^‡^P < 0.05 vs. OLETF-resistance training + 5α-reductase inhibitor group.

### GLUT-4 translocation and Akt and PKC-ζ/λ phosphorylation

**Fig 4:**
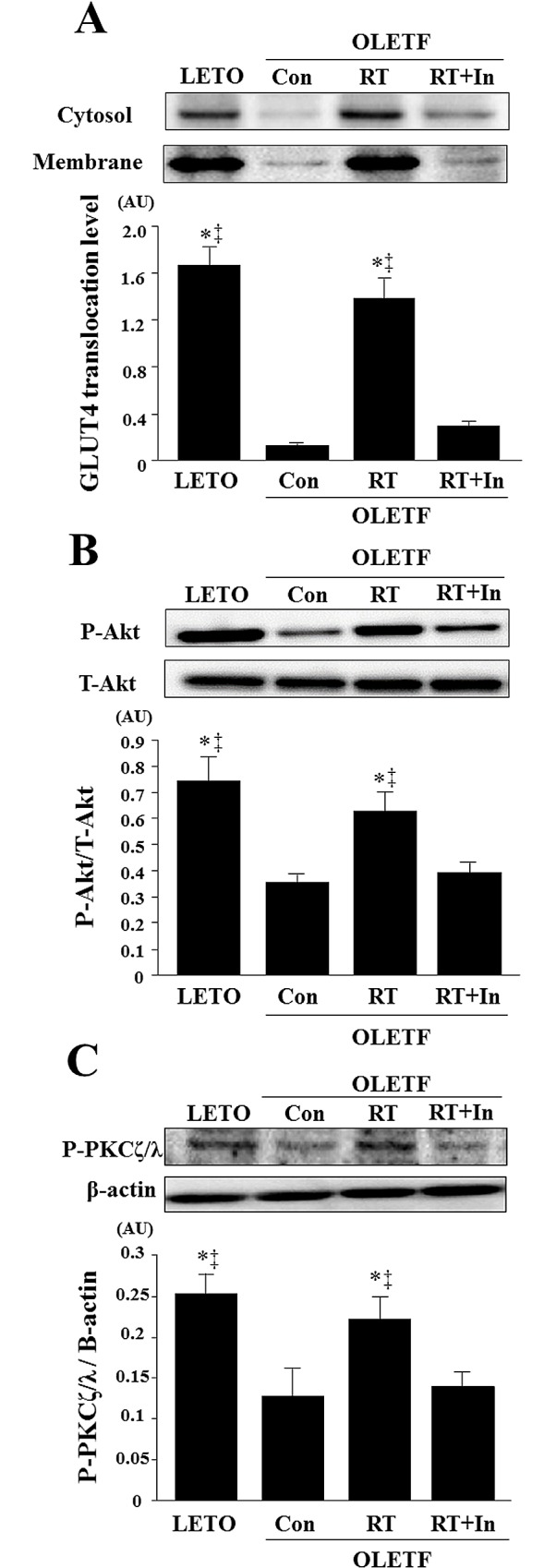
Effects of resistance training on GLUT-4 translocation (A) and phosphorylation of Akt (B) and PKC-ζ/λ (C) in gastrocnemius muscle. Representative immunoblotting images and histograms of GLUT-4 in cytosolic and membrane fractions, and levels of phospho-Akt, total Akt, phospho-PKC-ζ/λ, are shown. β-actin protein was employed as an internal control for normalization. AU, arbitrary units. Data are expressed as means ± SE. *P < 0.05 vs. OLETF-sedentary control group; ^‡^P < 0.05 vs. OLETF-resistance training group + 5α-reductase inhibitor group.

GLUT-4 translocation and Akt and PKC-ζ/λ phosphorylation levels were significantly lower in OLETF control rats than in LETO rats (Fig 4). In the resistance-trained OLETF rats, GLUT-4 translocation and Akt and PKC-ζ/λ phosphorylation levels were significantly elevated, but this was suppressed by chronic administration of the 5α-reductase inhibitor (Fig 4).

### Relationships among sex steroid hormones and glucose metabolism indices and muscle mass

Muscular DHT level was negatively correlated with fasting glucose level (r = -0.684, P < 0.05) and positively correlated with QUICKI (r = 0.571, P < 0.05), GLUT-4 translocation (r = 0.881, P < 0.05), gastrocnemius muscle mass (r = 0.701, P < 0.05), gastrocnemius CSA (r = 0.684, P < 0.05), soleus muscle mass (r = 0.648, P < 0.05), plantaris muscle mass (r = 0.597, P < 0.05), 5α-reductase protein expression (r = 0.826, P < 0.05), Akt phosphorylation level (r = 0.777, P < 0.05), and PKC-ζ/λ phosphorylation level (r = 0.649, P < 0.05). Furthermore, gastrocnemius muscle mass was negatively correlated with fasting glucose level (r = -0.806, P < 0.05) and positively correlated with QUICKI (r = 0.825, P < 0.05), GLUT-4 translocation (r = 0.865, P < 0.05), gastrocnemius CSA (r = 0.793, P < 0.05), 5α-reductase protein expression (r = 0.709, P < 0.05), Akt phosphorylation level (r = 0.783, P < 0.05), and PKC-ζ/λ phosphorylation level (r = 0.597, P < 0.05). Moreover, GLUT-4 translocation was negatively correlated with fasting glucose level (r = -0.758, P < 0.05) and positively correlated with QUICKI (r = 0.710, P < 0.05), gastrocnemius CSA (r = 0.689, P < 0.05), soleus muscle mass (r = 0.745, P < 0.05), plantaris muscle mass (r = 0.808, P < 0.05), 5α-reductase protein expression (r = 0.903, P < 0.05), Akt phosphorylation level (r = 0.837, P < 0.05), and PKC-ζ/λ phosphorylation level (r = 0.638, P < 0.05).

## Discussion

The results of this study demonstrated that chronic resistance training induced a significant increase in muscle tissue levels of DHEA, testosterone, and DHT; increased protein levels of steroidogenic enzymes; reduced fasting glucose level; improved insulin sensitivity index (QUICKI), and increased muscle mass and cross-sectional area in OLETF diabetic rats. The Akt/PKC-ζ/λ-GLUT-4 pathway was also upregulated in muscle after resistance training in the diabetic rats. Notably, resistance training-induced improvements in muscle mass and hyperglycemia with upregulation of GLUT-4-regulated signaling were suppressed by chronic inhibition of 5α-reductase. Furthermore, these changes in muscular DHT levels were significantly associated with muscle mass, fasting glucose level, insulin sensitivity index, and GLUT-4 translocation. Therefore, these results suggest that resistance training-induced elevations in the intramuscular synthesis of DHT were necessary to achieve improvement of hyperglycemia and muscle hypertrophy in type 2 diabetic rats.

In this study, resistance training in OLETF rats induced a significant increase in muscle tissue levels of DHEA, testosterone, and DHT and concomitantly, elevated the protein expression of androgen receptor and sex steroidogenic enzymes such as 3β-HSD, 17β-HSD, and 5α-reductase in skeletal muscles. DHEA is converted to testosterone by 3β- HSD and 17β-HSD, and testosterone is then converted to DHT by 5α-reductase [26]. Our previous study revealed that aerobic exercise training elevated the expression of these sex steroidogenic enzymes with increase in muscle DHEA and DHT levels in Zucker diabetic rats [16]. Thus, not only aerobic exercise training but resistance training as well may be effective to improve the reduced sex hormone production in skeletal muscle. Furthermore, the increased androgen receptor expression may enhance binding to androgenic hormones, testosterone, and DHT in the skeletal muscle in type 2 diabetes.

Several studies have shown a reduction in fasting glucose and HbA1c levels after resistance exercise intervention in type 2 diabetic patients [3–6]. Additionally, resistance training-induced increases in GLUT-4 translocation and protein expression of Akt and insulin receptor in the skeletal muscle participated in the improvement of glycemic control in type 2 diabetic patients and rats. [7, 27]. In the present study, similar to previous studies, the 8-week resistance-trained diabetic rats showed increased muscular GLUT-4 translocation (which affects the reduction of fasting glucose) as well as Akt and PKC-ζ/λ phosphorylation levels. Importantly, the resistance-trained OLETF rats that received the 5α-reductase inhibitor did not receive the beneficial effects of exercise on hyperglycemia or activation of GLUT-4-regulated signaling in the skeletal muscle. In cultured muscle cells, DHEA-derived DHT and testosterone activate GLUT-4 translocation and downstream signaling, and this activation is suppressed by treatment with a 5α-reductase inhibitor [12]. Thus, the increase in muscular DHT may contribute to the beneficial effects of regular resistance exercise on hyperglycemia and insulin sensitivity index in type 2 diabetes patients. Additionally, in this study, resistance training in OLETF improved insulin sensitivity index, as assessed by QUICKI, but did not change fasting insulin levels. Our previous study showed that aerobic exercise training reduced fasting insulin and glucose levels in diabetic rats [16]. Therefore, the effect of fasting insulin level may differ between aerobic and resistance exercise. Moreover, to clarify the effect of resistance training on insulin sensitivity, further study needs to be conducted to examine the physiological test such as a glucose and/or insulin tolerance test.

In this study, resistance training in OLETF rats significantly increased muscle mass and muscle CSA as well as muscular DHT level and expression of androgen receptor and sex steroidogenic enzymes such as 3β-HSD, 17β-HSD, and 5α-reductase. Our recent studies demonstrated that 12 weeks of resistance training improved age-related decreases in muscular DHT and sex steroidogenic enzyme expression and, coincidentally, resulted in a relationship between muscular DHT level and muscle size and strength in older men [15]. Testosterone administration increases muscle mass and may cause muscle fiber hypertrophy [28, 29]. In contrast, castration of male mice (effectively removing DHT synthesis) reduces muscle CSA through downregulation of protein synthesis [10]. In the present study, the diabetic rats that trained under administration of the 5α-reductase inhibitor showed decreased muscular DHT levels and a subsequent suppression of training-induced muscle hypertrophy. Furthermore, muscle DHT levels were correlated with muscle mass and the effects of training. Therefore, muscular DHT synthesis relates to muscle hypertrophy in response to chronic resistance training in type 2 diabetes. Furthermore, in this study, the resistance training-induced increase in gastrocnemius CSA and LV mass in OLETF rats were not completely suppressed by chronic inhibition of 5α-reductase. Since other mechanisms as well as sex steroid hormones are involved in the increasing skeletal muscle mass by resistance training, it is considered that this increase could not be completely suppressed. Further study needs to examine other molecular mechanism.

Muscle mass and strength is lower in patients with type 2 diabetes compared to healthy individuals [8, 30]. Additionally, low muscle mass adversely affects insulin resistance [31] and may be a predictor of diabetes incidence [32]. In contrast, an increase in muscle mass is associated with reduced insulin resistance, which can be estimated by the homeostasis model assessment of insulin resistance (HOMA-IR) [10]. In this study, chronic resistance exercise induced muscle hypertrophy and improved fasting glucose level and insulin sensitivity index in type 2 diabetic rats. Moreover, resistance training-induced muscle hypertrophy was correlated with the training effects of fasting glucose level and QUICKI. Therefore, muscle hypertrophy due to regular resistance exercise may partly contribute to improvement of glycemic control. Additionally, the beneficial effects of resistance training on muscle mass and hyperglycemia were suppressed by chronic inhibition of 5α-reductase. Thus, this study suggests that increased skeletal muscle sex steroid hormone levels may improve hyperglycemia due to increases in muscle mass.

Our previous study revealed that the basal mRNA expression of steroidogenic enzymes, i.e., 3β-HSD and P450arom, are differentially expressed in different skeletal muscle type, but 17β-HSD mRNA expression does not change [11]. In this study, resistance training increased protein expression of 3β-HSD, 17β-HSD, and 5α-reductase in the gastrocnemius muscle of type 2 diabetic rats. Gastrocnemius muscle comprises a mixture of type I and type II muscle fibers. Therefore, further studies are essential to examine whether the responses to resistance training differ between slow- and fast-twitch fibers of the muscle.

In conclusion, habitual resistance exercise in type 2 diabetic rats increased muscular sex steroid hormone levels and concomitantly induced muscle hypertrophy and improved hyperglycemia and insulin sensitivity index by activating GLUT-4–regulated signaling in skeletal muscle. Chronic administration of a DHT synthesis inhibitor suppressed the beneficial effects of resistance training. Therefore, these results suggest that resistance training-induced elevations in muscular DHT were necessary to improve hyperglycemia and cause muscle hypertrophy in type 2 diabetic rats. Thus, regulating muscular DHT is a potential new therapy strategy for diabetes.
